# Supplementary material for: Normal Ovarian Function in Subfertile Mouse with Amhr2-Cre-Driven Ablation of Insr and Igf1r
Source: Genes (Basel). 2024 May 12;15(5):616. doi: 10.3390/genes15050616 (PMC11121541; doi:10.3390/genes15050616)
Supplement: Supplementary file 1 [file genes-15-00616-s001.zip › genes-2997443-supplementary.pdf]

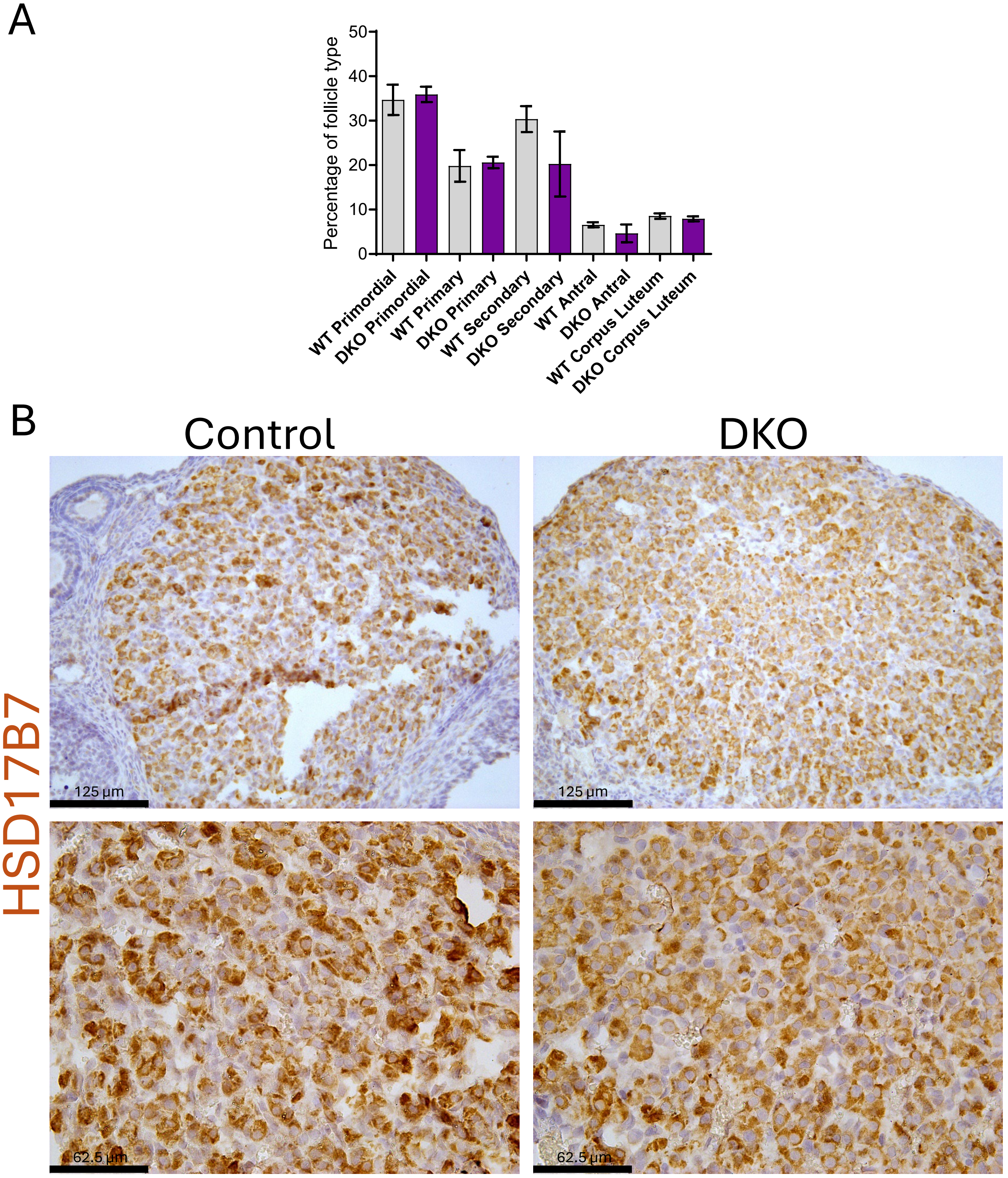

Supplemental Figure S1. Histological analysis of folliculogenesis and luteinization in *Amhr2*-Cre *Insr* and *Igf1r* DKO mice. (**A**) Ovaries were serially sectioned and total follicle numbers counted. Data are expressed as mean ± SEM percentage of each follicle type in the total follicle pool. (**B**) Immunohistochemistry localization of the luteal cell marker HSD17B7. No oocyte retention in WT or DKO mice was observed in *Amhr2*-Cre propagated mutants as was prevalent in *Pgr*-Cre mediated deletions of both *Insr* and *Igf1r*.

Table S1. PCR Primers used for analysis of *Amhr2*-Cre insulin receptor knockout mice

Genotyping Primers

Gene		Sequence 5'-3'	Length (bp)	TM
Insr-flox	For<br>Rev	GAT GTG CAC CCC ATG TCT G<br>CTG AAT AGC TGA GAC CAC AG	279 (WT)<br>313 (mutant)	62
Igf1r-flox	For<br>Rev	CTT CCC AGC TTG CTA CTC TAG G<br>CAG GCT TGC AAT GAG ACA TGG G	124 (WT)<br>220 (mutant)	62
Amhr2-Cre	Amhr2-For<br>Amhr2-Rev<br>Cre-For<br>Cre-Rev	AGG TGG GTC AGA CCC AGA GCC<br>GCA TGA CCT CCT TCC TGG ATT<br>CCG CTT CCT CGT GCT TTA CGG TAT<br>ACC TAG TAG AGA GGC TGC GTT GA	223 (WT)<br>500 (Cre)	66

Primers used for qPCR

Accession number	Gene		Sequence of forward and reverse primers 5'-3'
	Lhcgr	For<br>Rev	GAGACGCTTTATTCTGCCATCT<br>CAGGGATTGAAAGCATCTGG
	Pgr	For<br>Rev	CTCCGGGACCGAACAGAGT<br>ACAACAACCCTTTGGTAGCAG
	Ptgs2	For<br>Rev	CTGACCCCCAAGGCTCAAAT<br>ATTTAAGTCCACTCCATGGCCC
	Star	For<br>Rev	GAAAGCCAGCAGGAGAACG<br>GCGGTCCACAAGTTCTTCAT
	Cyp11a1	For<br>Rev	TGTGATTTTCAATAAAGCTGATGA<br>TTCTTGAAGGGCAGCTTGTT
	Cyp17a1	For<br>Rev	TTTATGCCTGAGCGCTTCTT<br>GCAGCAAGGCCATGAAGATA
	Cyp19a1	For<br>Rev	CTGTTGTGGGTGACAGAGACA<br>GCCGTCAATTACGTCATCCT
	Hsd3b1	For<br>Rev	AACAATTTAACAGCCCTCCTAAG<br>GCACCAACATCTTGATGATCC
	Hsd17b1	For<br>Rev	AGGTGACGGAGCTCTTCTTG<br>CGACATAGCTGCTGCCACT
	Hsd17b7	For<br>Rev	GGGCCAAAAGATGGACATAG<br>AGGAACGCCTACATCAGCTC
	Rpl19	For<br>Rev	TGCCTCTAGTGTCCTCCGC<br>ATCCGAGCATTGGCAGTACC
